# Preparation from a revisited wet chemical route of phase-pure, monocrystalline and SHG-efficient BiFeO_3_ nanoparticles for harmonic bio-imaging

**DOI:** 10.1038/s41598-018-28557-w

**Published:** 2018-07-11

**Authors:** Gareth Clarke, Andrii Rogov, Sarah McCarthy, Luigi Bonacina, Yurii Gun’ko, Christine Galez, Ronan Le Dantec, Yuri Volkov, Yannick Mugnier, Adriele Prina-Mello

**Affiliations:** 1 Department of Clinical Medicine, Trinity Translational Medicine Institute (TTMI), Trinity College Dublin, Dublin 8, Ireland; 2 CRANN Institute and AMBER centre, Trinity College Dublin, Dublin 2, Ireland; 3 Univ. Savoie Mont Blanc, SYMME, F-74000 Annecy, France; 4 GAP – Biophotonics, Université de Genève, 22 Chemin de Pinchat, CH-1211 Genève 4, Switzerland; 5 School of Chemistry, Trinity College Dublin, Dublin 2, Ireland; 6 Department of Histology, Cytology and Embryology, First Moscow State Sechenov Medical University, Moscow, Russian Federation

**Keywords:** Nonlinear optics, Photonic crystals

## Abstract

We present two new synthetic routes for bismuth ferrite harmonic nanoparticles (BiFeO_3_ HNPs). Both phase-pure and mixed phase BiFeO_3_ materials were produced after improvement of the solvent evaporation and sol-gel combustion routes. Metal nitrates with a series of dicarboxylic acids (tartronic, tartaric and mucic) were used to promote crystallization. We found that the longer the carbon backbone with a hydroxyl group attached to each carbon, the lower the annealing temperature. We also demonstrate that nanocrystals more readily formed at a given temperature by adding glycerol but to the detriment of phase purity, whereas addition of NaCl in excess with mucic acid promotes the formation of phase-pure, monocrystalline nanoparticles. This effect was possibly associated with a better dispersion of the primary amorphous precursors and formation of intermediate complexes. The nanoparticles have been characterized by XRD, TEM, ζ-potential, photon correlation spectroscopy, two-photon microscopy and Hyper-Rayleigh Scattering measurements. The improved crystallization leads to BiFeO_3_ HNPs without defect-induced luminescence and with a very high averaged second harmonic efficiency (220 pm/V), almost triple the efficiency previously reported. This development of simple, scalable synthesis routes which yield phase-pure and, crucially, monocrystalline BiFeO_3_ HNPs demonstrates a significant advance in engineering the properties of nanocrystals for bio-imaging and diagnostics applications.

## Introduction

Bismuth ferrite is a very important material due to its magnetoelectric, piezoelectric and nonlinear optical properties^1–8^. Recent advances in nanoscale ferroelectrics have renewed and accelerated worldwide interest in BiFeO_3_ because it is a near-ideal candidate for applications in fields as diverse as energy harvesting^9^, non-volatile memory^10–12^, spintronics^13,14^ and nonlinear optics^7,15,16^. BiFeO_3_ is a highly ferroelectric, multiferroic material with a rhombohedrally distorted perovskite structure belonging to the R3c space group^2^. The noncentrosymmetry arises from the rotation of the oxygen octahedrons around the pseudocubic [111] axis^3^, as shown in the Supplementary Fig. S1. BiFeO_3_ displays antiferromagnetic ordering below its Néel temperature that ranges between 310 and 370 °C according to the crystallite size^1,17,18^.

The noncentrosymmetric structure of BiFeO_3_ enables even-order nonlinear optical responses such as second harmonic generation (SHG). SHG occurs when two photons of one frequency are combined in a crystal, resulting in a single photon of exactly double the input frequency. Because harmonic generation is a non-resonant process, the excitation can be tuned to any frequency within the crystal’s transparency range^19–23^. Exploiting this phenomenon for bio-imaging has further advantages over existing techniques. For example, fluorescent moieties bleach and quantum dots may blink. In contrast, harmonic generation in a nonlinear optical nanocrystal or Harmonic Nanoparticle (HNP) allows long term observation^20,24,25^. The ability to tune the input (and hence also the output) frequency is very significant for biomedical applications – frequencies can be tuned to avoid sample absorbance and to prevent energy deposition in the sample. Applications in the biomedical field include deep-tissue imaging with second but also third harmonic, for instance, as an unmet clinical challenge^26–28^. To this end, the appealing optical and magnetic properties of BiFeO_3_ HNPs combined with its low cytotoxicity^29^ have motivated this work.

Applications using BiFeO_3_ HNPs are however still limited by the difficulty in determining a facile, scalable synthesis route which yields nanoscale, monodisperse and monocrystalline nanoparticles. Conventional solid-state processing is restricted by inherent thermodynamic and kinetic aspects, as recently discussed^30,31^. Phase purity, size control and dispersion are also a significant challenge often mentioned in the literature for the many other existing routes that include hydrothermal, sol-gel combustion, sonochemical and microemulsion techniques^32–46^. As preparation of monocrystalline harmonic diagnostic-carriers are here foreseen, we further develop the most promising wet chemical routes, namely Pechini’s sol-gel method and Ghosh’s solvent evaporation approach^47^. Much research has been done to determine the best chelating agent for use as a template, particularly by Selbach *et al*.^48^ Carboxyl groups are required for complexing the Bi^3+^ and Fe^3+^ metal ions to obtain a homogeneous polyester precursor whereas hydroxyl groups are necessary for the subsequent polyesterification of the carboxyl groups. The choice of chelating and complexation agents has however a considerable impact on the phase-purity, size distribution and crystallinity of the products for a given annealing temperature. In the current study, a variety of dicarboxylic acids allowing a systematic variation of the ratio of hydroxyl groups to carboxyl groups from 1:2 to 2:1 is investigated with tartronic acid, tartaric acid and mucic acid, respectively. So far, syntheses with the tartronic and mucic acids have not been reported. The lowest crystallisation temperature being obtained with mucic acid and with the aim to further inhibit Ostwald ripening, the synthesis is then refined by addition of glycerol and NaCl acting as a spacer before the crystallization step. The key influence of NaCl and extra hydroxyl groups on the final nanoparticle size, polydispersity and crystallinity is investigated through XRD, TEM, magnetic measurements and assessment of the functional SHG response.

## Results and Discussion

### Effect of chelating agent on the crystallization temperature

**Figure 1:**
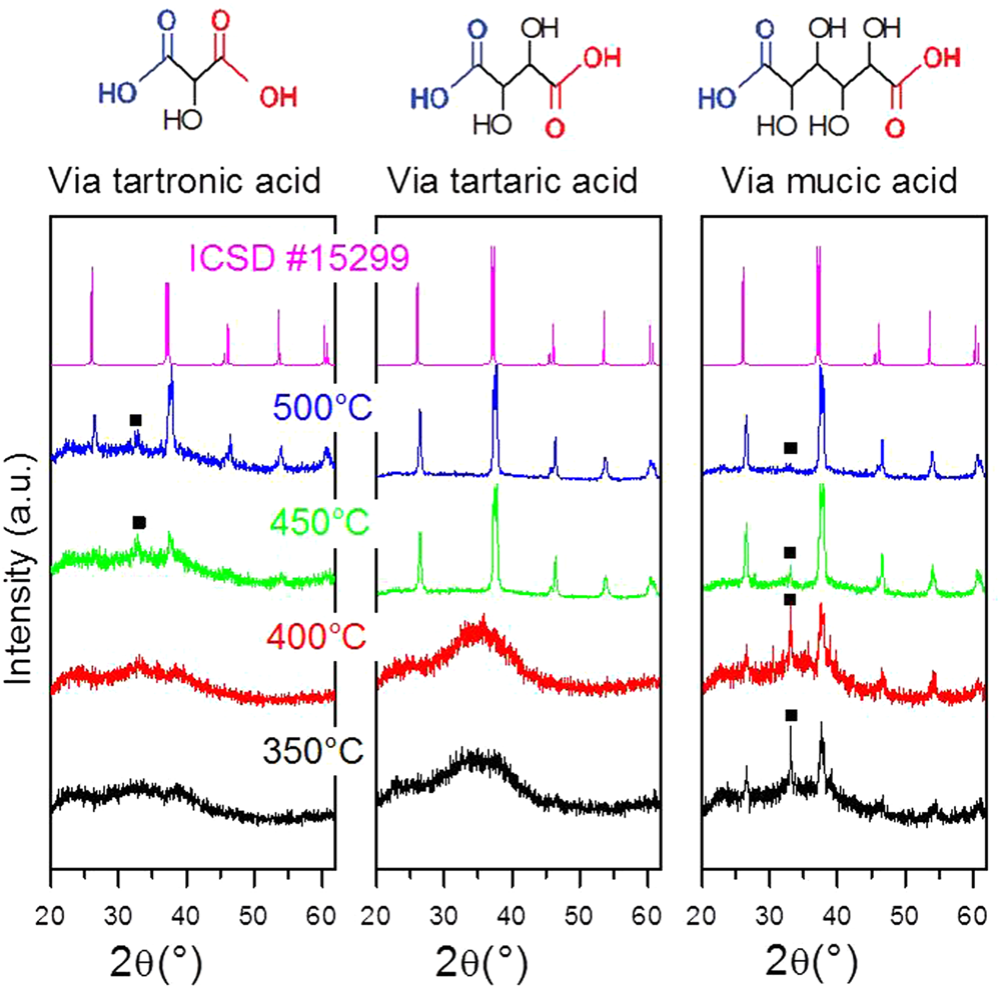
Structure of chelating agents of increasing length and increasing ratio of hydroxyl to carboxyl groups (top). Corresponding temperature-resolved XRD patterns of BiFeO_3_ synthesized (bottom) – left, tartronic acid; middle, tartaric acid; right, mucic acid. The longer the carbon backbone, the lower the temperature required to form bismuth ferrite, albeit with phase impurities. The BiFeO_3_ reference pattern corresponds to ICSD #15299 and the extra peak denoted by ■ at 2θ ≈ 32.2° belongs to the Bi_25_FeO_39_ phase.

BiFeO_3_ HNPs were synthesized with chelating agents of increasing length and increasing ratio of hydroxyl groups to carboxyl groups, such as tartronic (1:2), tartaric (1:1) and mucic acid (2:1), resulting in different annealing temperatures required to form phase-pure nanoparticles as can be seen from the temperature-resolved XRD patterns in Fig. 1.

For all chelating agents, the evaporation of the solvent was accompanied by the evolution of NO_x_. However, in the case of mucic acid, an orange gel formed as the last of the solvent evaporated, accompanied by a rapid increase in volume. With continued heating and stirring, the gel turned to powder and changed colour to light greenish brown and when left on the hotplate at 160 °C the powder finally combusted to form a dark brown powder. The combustion produced no flame as in other combustion methods^38,42^; it merely smouldered. Temperature-dependent XRD analysis showed that a phase impurity, namely Bi_25_FeO_39_, and a significant amorphous background are introduced below 450 °C when the sample is allowed to combust in this manner. Similar results were already obtained^48^ when tartaric acid was used as the chelating agent in the presence of ethylene glycol during annealing. The lowest crystallization temperature to form BiFeO_3_ with the solvent evaporation route (for the tartronic and tartaric acids) and with the combustion method (for the mucic acid) is summarized in Table 1 according to the increasing -OH: -COOH ratio.

**Table 1:** Ratio of hydroxyl groups to carboxyl groups and lowest crystallisation temperature to form BiFeO_3_ for each of the chelating agents used in this study. With mucic acid, the sample ignited as soon as the solution had evaporated at approximately 160 °C.

Chelating agent	Tartronic acid	Tartaric acid	Mucic acid
-OH:-COOH	1:2	1:1	2:1
Lowest annealing temperatura (30 min annealing)	>500 °C	<450 °C	<350 °C

Upon reaching 350 °C, BiFeO_3_ becomes the dominant phase.

Ghosh *et al*.^47^ suggested that tartaric acid yields phase-pure bismuth ferrite because it forms polymeric precursors as opposed to dimeric precursors as in the case of citric acid. Bidentate interaction of Bi^3+^ with carboxylate/deprotonated hydroxyl groups of four tartarate ligands indeed lead to the formation of heterometallic polynuclear complexes in solution where the two metal ions are homogeneously dispersed throughout the network. The longer carbon backbone of mucic acid makes the molecule more flexible and prone to coordinate with bismuth and iron ions in solution in a polymeric array. This leads, together with the combustion step induced by the higher content of hydroxyl groups, to crystalline BiFeO_3_ even at temperatures as low as 350 °C.

A comparison may be drawn again here to the work of Selbach *et al*.^48^. In their paper, other dicarboxylic acids of varying chain length were investigated, namely malic, maleic, malonic and succinic acid. Their results also indicated that for BiFeO_3_ precursors to form, carboxyl groups are required for complexing the metal ions and that hydroxyl groups are required for the polyesterification of carboxyl groups, whether these were present on the chelating molecule (for the malic acid) or as alcohol in solution. Our results suggest that the more carboxylate/deprotonated hydroxyl groups available during formation, the lower the temperature required to form BiFeO_3_. It is important to note that although BiFeO_3_ was present as the dominant phase in the mucic acid sample at 350 °C, higher temperatures are required to rid the sample of residual impurities. To further investigate the effect of hydroxyl groups, refinement was then carried out on mucic acid and the ratio of -OH:-COOH was further increased by adding glycerol.

### Additional -OH groups increase crystallinity at lower temperatures

Temperature-resolved XRD analysis depicted in the Supplementary Fig. S2 shows that phase impurities are more readily introduced when the sample is allowed to combust with glycerol, similar to the results of Selbach *et al*.^48^ when tartaric acid was used as the chelating agent and annealed in the presence of ethylene glycol. Here, initial addition of 2 mmol of glycerol further increased the ratio of hydroxyl to carboxyl groups to 3.5:1. Comparing the height of the XRD peaks to the height of the amorphous background, additional -OH groups increased the crystallinity of any phases present at lower temperatures. If the size of the largest peaks relative to the amorphous background is taken as a measure of how well-crystallized the nanoparticles are, at 500 °C the presence of glycerol has no significant impact on the crystallinity, whereas at 200 °C, there is significant enhancement as can be seen in Fig. S2. Adding glycerol thus lowers the crystallization temperature but promotes the formation of the Bi_25_FeO_39_ and of an intermediary oxide phase. An annealing temperature above 500 °C is then needed to obtain phase-pure BiFeO_3_.

**Figure 2:**
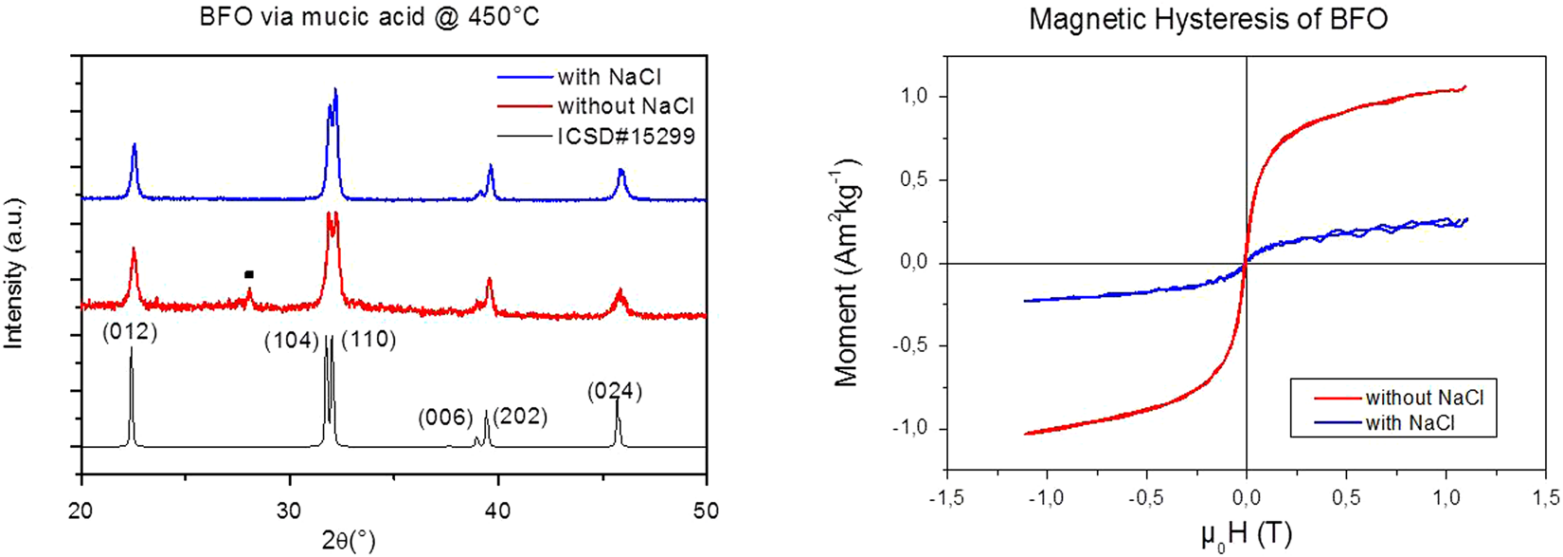
(left) Room temperature XRD patterns of BiFeO_3_ powders prepared without (red) and with (blue) NaCl added to the precursors. Note that combustion was not allowed in each synthesis and organics were further removed in the drying and baking steps for 2 h at 140 °C and 350 °C, respectively. Peak denoted by ■ belongs to the Bi_25_FeO_39_ phase (right) Corresponding Vibrating Sample Magnetometry measurements of BiFeO_3_ prepared with and without NaCl. XRD and magnetic measurements indicate that NaCl promotes phase purity.

If the presence of hydroxyl groups is actually necessary for the subsequent polyesterification of the carboxyl groups, additional -OH groups in the mucic acid route mostly promotes the gel combustion after the solvent evaporation step. Because the Bi_25_FeO_39_ and Bi_2_Fe_4_O_9_ phases are slightly more thermodynamically stable than BiFeO_3_ in the temperature range 447 °C–767 °C^30,31^ a non-uniform temperature or ill-defined local thermodynamic conditions during the combustion very likely account for the formation of mixed phase products containing the intermediary Bi_25_FeO_39_ phase. Similarly, impurities are also produced when ethylene glycol is added as a polymerizer with tartaric acid and if the combustion is not prevented with an extra baking step below 200 °C^48^. It is worth to mention that such an additional baking step was also proven to be vitally important if phase-pure powders are desired at lower temperatures with mucic acid. This can be achieved by removing the beaker from the furnace at 140 °C as soon as the gel begins to form and allowing the remaining nitrates to evaporate for 2 hours. Upon heating, when the temperature is raised to 300–350 °C for an extra baking step of 2 h, a colour change is observed after final elimination of the remaining organic compounds. An example of an almost phase-pure sample then annealed at 450 °C is shown in Fig. 2. Without any additional OH groups, this represents a significant improvement in the lowest calcination temperature reported by Ghosh *et al*. (~500 °C) for well-crystallised BiFeO_3_ HNPs articles via tartaric acid.

On the other hand, the mean nanocrystal size of BiFeO_3_ estimated from the Supplementary Fig. S2 with Scherrer’s formulae is continuously increased from ~20 to ~50 nm as the calcination temperature is raised from 200 to 500 °C. Such a size increase evidences the Ostwald ripening process and the continuous chemical reaction between the primary seeds, impurity phases and remaining amorphous precursors. As above, the synthesis clearly fails in the preparation of well-dispersed monocrystalline nanoparticles. In the next section, addition of an excess of salt (NaCl) as a spacer is originally introduced with the aim to better homogenise the reactive medium during the evaporation step and to limit as much as possible aggregation and formation of intermediary phases that both evidently inhibit the direct formation of monocrystalline particles of controllable size and shape.

### NaCl promotes crystal formation and phase purity

An excess of NaCl (10 mmol) was dissolved in the mucic acid solution prior to evaporating the solvent. The annealing of amorphous precursors was then carried out as before (combustion is prevented by a 2 h drying step at 140 °C, whereas remaining organic compounds are then removed with the 2 h extra baking step at 350 °C) except that they were dispersed in the salt matrix to reduce the Ostwald ripening process. Interestingly, the presence of salt not only lowers the crystallisation temperatures but also results in better crystallized phase-pure powders. For comparison, Fig. 2 shows the XRD patterns of BiFeO_3_ prepared with and without NaCl all annealed at 450 °C. The presence of NaCl in excess clearly enhances the phase purity and degree of crystallization. From the XRD diffraction pattern, the absence of any residual impurity and amorphous background can indeed be noticed.

The high degree of purity and crystallinity is very consistent with the room temperature magnetic response measured from Vibrating Sample Magnetometry (VSM). BiFeO_3_ HNPs only display weak ferromagnetism and a well-known size-dependent saturation magnetization (Ms)^49,50^. Interestingly, Ms values have been found below ~0.6 Am^2^/kg for nanocrystals within the 18–83 nm size range prepared from tartaric acid^51^. Here, without NaCl, the mean size estimated from the Scherrer’s formulae is ~50 nm so that the high Ms value at about 1.03 Am^2^/kg can only be attributed to γ-Fe_2_O_3_ or Fe_3_O_4_ parasitic impurities as recently also observed when tartaric acid is used in the solvent evaporation route^52^ or when combustion is allowed to occur^15^. Note that in both cases the magnetic impurities were hardly detected from standard XRD measurements. Interestingly, the Ms value at 1T dropped at ~0.23 Am^2^/kg for nanocrystals produced with an excess of NaCl. This latter saturation magnetization value is in complete agreement with the one already reported for pure BiFeO_3_ HNPs of similar size^51,52^ showing no trace of magnetic impurity.

**Figure 3:**
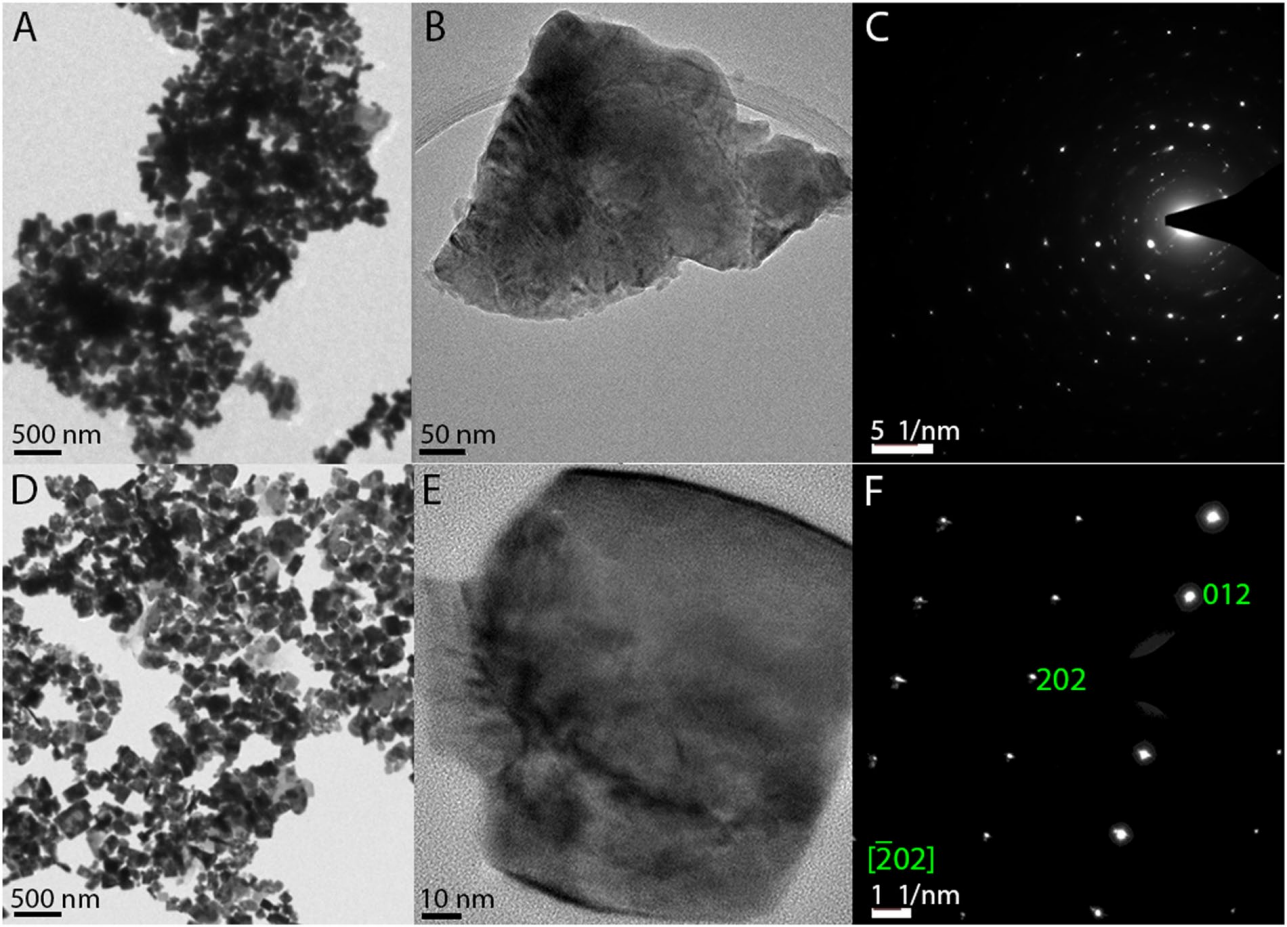
TEM, HRTEM and SAED patterns showing that NaCl with mucic acid leads to monocrystalline NPs. (**A**) TEM image showing panoramic view of aggregated BiFeO_3_ HNPs prepared without NaCl and (**B**) corresponding HRTEM image showing an amorphous surface; (**C**) SAED pattern of the individual nanoparticle in B, showing diffraction spots of BiFeO_3_ but from multiple crystal domains. (**D**) TEM image showing panoramic view of aggregated BiFeO_3_ NPs prepared with NaCl and (**E**) corresponding HRTEM image showing the lattice spacings of well-crystallized BiFeO_3_ HNPs; (**F**) SAED pattern of the individual nanoparticle in E, showing diffraction spots indicating that the HNP is monocrystalline.

Because of the frequently observed size- and impurity- dependent magnetic responses of BiFeO_3_ HNPs, TEM measurements were performed and they were found consistent with the above VSM and XRD data. From the high resolution TEM and Selected Area Diffraction (SAED) images shown in Fig. 3, it can be seen that particles prepared without salt have an amorphous surface layer (as well as trace impurities of Bi_25_FeO_39_). This can only be attributed to the surface which is amorphous because the selected area electron diffraction patterns, inset, indicate a high degree of crystallinity even in the sample whose lattice spacings could not be imaged (top row). However it is also apparent from the visible rings and the large number of spots in the SAED that the sample is polycrystalline which can also contribute to an increase of the weak ferrogmatism^53^.

By contrast, the HRTEM and SAED patterns of individual nanoparticles prepared via NaCl showed the samples to be monocrystalline as the same crystal lattice extends throughout the particles and the spot pattern is a clean array, without amorphous rings or multiple scatterings.

One explanation for the increased crystallinity may be related to a better dispersion of the primary amorphous precursors within the salt matrix. The presence of intermediary salts due to side reactions, for instance Fe(ClO_4_)_3_⋅9H_2_O and FeCl_3_⋅6H_2_O that are known to catalyse esterification^54^, might also provide a lower energy pathway. Greater concentration of salt may also permit more salt bridges to form between the hydroxyl and carboxylate groups thus promoting the formation of the polymeric precursor.

Having established the capability to scale-up and produce large amounts (>1 g per batch) of monocrystalline BiFeO_3_ HNPs, the nonlinear optical properties were then studied as another indicator of their enhanced properties by probing dispersions of varying concentration.

### Improved crystallization enhances second harmonic properties

**Figure 4:**
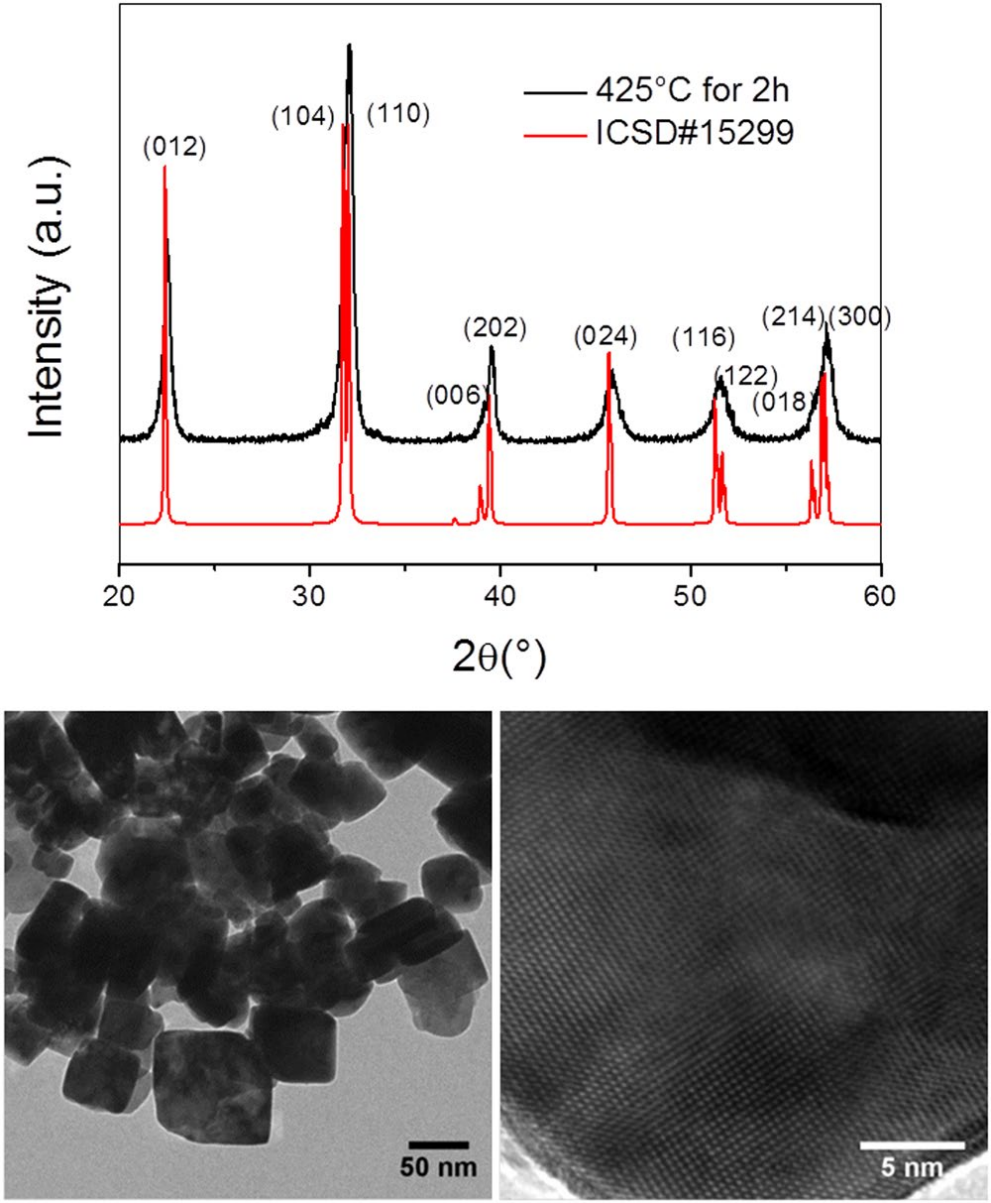
XRD diffraction pattern and corresponding TEM images of BiFeO_3_ synthesised via the mucic acid route using NaCl and annealing at 425 °C for two hours.

After optimization of the solvent evaporation route without combustion and with mucic acid as a chelating agent, we synthesised phase-pure, monocrystalline BiFeO_3_ HNPs by annealing for 2 hours at 425 °C in NaCl. 425 °C was found to be the lowest temperature to obtain phase-pure powders without indication of an amorphous background as shown from the XRD diffraction pattern in Fig. 4. The mean XRD size estimated at ~40 nm is found very consistent with the TEM imaging.

For a quantitative assessment of the Second Harmonic (SH) properties, it was necessary to obtain stable colloidal dispersions. After dispersion of desired amount of powders, sedimentation of residual aggregates was observed by time-resolved Dynamic Light Scattering as well as by the change in the opaqueness of the sample over two days across the pH range investigated (see the Supplementary Figs S3 and S4). Once a colloidal suspension of sufficient stability and monodispersity has been achieved, the nonlinear optical response was determined by Hyper-Rayleigh Scattering (HRS) measurements as described elsewhere^55^. The variation of HRS intensity with concentration is shown in the Supplementary Fig. S5. This result was used to determine a second harmonic efficiency of 220 pm/V with a typical 15% uncertainty^56^. Comparing this result to a value of 7.4 pm/V measured with LiNbO_3_ NPs using the same setup^55^, it is clear that BiFeO_3_ is a very promising candidate for a 1064 nm wavelength excitation, partly because of the resonant character of its susceptibility around 504 nm as evidenced with spectroscopic ellipsometry^7^. This also constitutes a significant improvement in the SH efficiency of BiFeO_3_ NPs; a previously reported study carried out on the same setup analysing BiFeO_3_ NPs (synthesized via sol-gel combustion using TRIS as a starting fuel) determined the averaged <d> coefficient to be 79 pm/V^15^, further corroborating the conclusion that the synthesis route described here leads to much more monocrystalline, monodisperse and phase-pure nanoparticles.

**Figure 5:**
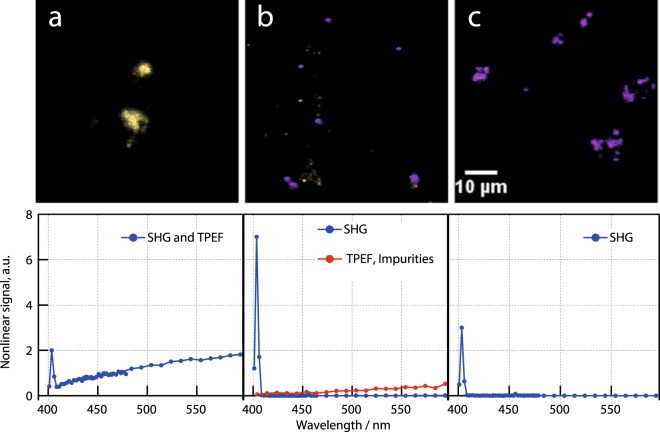
Second Harmonic Microscopy images of BiFeO_3_ nanoparticle aggregates immobilized in agarose (top row), with the corresponding two-photon emission spectra (bottom row): (**a**) using mucic acid without NaCl or glycerol but where combustion was prevented; (**b**) using mucic acid with NaCl and glycerol and with the unavoidable combustion step; and (**c**) using mucic acid and NaCl where combustion was prevented. Each image is a composite of 25 colours (from violet to red, corresponding to idenpendent detection channels): excitation was at 810 nm. Violet represents second harmonic (405 nm), white corresponds to the mix of other colours and represents two-photon excited luminescence. For sample b, the two-photon excited fluorescence (TPEF) signal acquired in the squared Region of interest (ROI) is plotted below in red in the corresponding spectrum.

Finally, the interplay between the synthesis conditions and the resulting optical properties for bioimaging is further illustrated below. Phase-purity, colloidal stability and monocrystallinity are crucial to the use of such materials because their SH and third harmonic properties are in practise far easier to model, in terms of brightness and optical contrast, than for mixed phase, polycrystalline or polydisperse materials^55^. In addition, uniqueness of the inherent, specific optical signatures of harmonic emission can be dramatically lost if a defect-related intense luminescence results from the chemical preparation. The significant improvement in the crystallinity and optical properties of BiFeO_3_ HNPs produced with NaCl is again demonstrated in Fig. 5 by use of a multiphoton optical imaging platform. Instead of simply filtering the optical emission, as usually, with interference filters centred at the SH wavelength, special attention has been paid to obtain spectrally resolved images in the range 400–600 nm. Bare BiFeO_3_ NPs were here dispersed in saline solution and subsequently deposited in 0.2% agarose gel to create a representative tissue mimicking phantom model for SHG imaging.

Note that particle size distributions and aggregation state affect the intensity of SH images so, contrary to previous HRS measurements, microscopy is here less prone to give reliable SH efficiencies, especially without a detailed analysis of the polarization-resolved measurements^15,16^. However, when comparing the SH microscopy images to the TEM observations above, it can be seen that the presence of NaCl promotes the formation of high-quality monocrystalline nanoparticles. Sample a (Fig. 5), where neither NaCl nor glycerol was used, shows an SH signal almost entirely occluded by two-photon excited luminescence. Some luminescence was observed in sample b (Fig. 5) where NaCl and glycerol were used, but it did not necessarily co-localize with the SH, suggesting that defects/impurities are not homogeneously dispersed in the sample and that the SH signal is stronger than luminescence for only a few ROI. By contrast, there was no stray two-photon excited luminescence when the sample was prepared using mucic acid and NaCl (Fig. 5, sample c), further indicating that the defect-induced luminescence (as seen in Fig. 5, samples a and b) is to be related to the growth and crystallization conditions. During the calcination step, Pechini sol-gel process is indeed well known to promote the incorporation of carbon impurities, oxygen defects as well as carbon dioxide and peroxyl radicals in various oxides^57^. To the best of our knowledge, such defects have not been specifically investigated in BiFeO_3_ yet and will constitute matter for a future work. However, it can be noticed though that the luminescence spectra in Fig. 5 (for the (a) and (b) samples) are very similar to the ones observed in amorphous perovskite materials like BaTiO_3_, PbTiO_3_ and SrTiO_3_^58^. An intense luminescence between 500 nm and 700 nm under near UV excitation was indeed observed for these amorphous particles prepared with citric acid as chelating agent, ethylene glycol as polymerizer and after calcination at 300–400 °C. Such a luminescence arising from amorphous structures is here very consistent with our XRD measurements and TEM observations.

## Conclusion

Within the framework of a multidisciplinary, pan-European study, significant improvements in both the solvent evaporation and sol-gel combustion routes have been demonstrated so as to scale up and produce high amounts of monocrystalline, phase-pure and defect-free BiFeO_3_ Harmonic NanoParticles (HNPs). The strong interplay between the increasing length of different chelating agents, ratio of hydroxyl to carboxyl groups and the addition of extra -OH groups have been related to the lowest necessary crystallisation temperature to prepare phase-pure samples. More originally, the use of NaCl as a spacer and catalyst before the calcination step led below its melting temperature may also open new chemical approaches for controlling the size and morphology of the as-synthesized nanoparticles. In the future, other salts could be used to further investigate their catalytic behaviour and the detailed mechanisms of their influence on the formation of highly monocrystalline nanomaterials with enhanced physicochemical properties. This is of importance in the biomedical field where exploring the advanced imaging potential of bright, harmonic nonlinear optical nanocrystals could lead to the development of future diagnostic products.

## Methods

### BiFeO_3_ synthesis

0.313 g of BiFeO_3_ HNPs have been prepared per batch by modifying Ghosh *et al*.’s method which is based on solvent evaporation^47^. Briefly, Bi(NO_3_)_3_⋅5H_2_O (0.485 g, 1 mmol) was dissolved in 2 M HNO_3_ (100 ml), followed by addition of Fe(NO_3_)_3_⋅9H_2_O (0.404 g, 1 mmol). In some cases, 2 mmol of chelating agent (see below) was also added, then the solution was heated under stirring at 165 °C for approximately 1 hr, until the solvent had evaporated. It is vitally important to prevent the gel from combusting if phase-pure powders are desired at lower temperatures. This can be achieved by removing the beaker to a furnace at 140 °C as soon as the gel begins to form and allowing the remaining nitrates to evaporate for 2 hours. Organics were then removed by baking at 350 °C for a further 2 hours. For the temperature dependent studies the powder was collected and then annealed at temperatures starting from 200 °C for periods of 30 minutes at intervals of 50 °C at 2 °C per minute, with a rest of 30 minutes after each ramp before XRD diffraction patterns were recorded.

A variety of chelating agents at 2 mmol were used: tartronic acid (0.238 g), tartaric acid (0.296 g) and mucic acid (0.412 g), which are of increasing length with increasing number of hydroxyl groups along their carbon chain. Glycerol was added to determine whether the ratio of hydroxyl groups to carboxyl groups had an influence on the crystallization of BiFeO_3_. In order to establish the optimal ratio of hydroxyl to carboxyl groups, the above protocol was repeated with mucic acid, but glycerol (0.184 g, 2 mmol) was added to the solution before evaporating, yielding a final -OH:-COOH ratio of 3.5:1.

To promote crystallization and to inhibit Ostwald ripening, the mucic acid procedure was also carried out in the presence of salt by dissolving excess NaCl (0.585 g, 10 mmol) in the solution prior to evaporating the solvent.

After annealing, powders were washed four times in ethanol and Millipore water.

### Characterisation

Structure analysis was carried out by XRD (using Co Kα radiation from an INEL CPS 120 for the temperature-dependent XRD studies, and with Cu Kα radiation using a Philips X’pert PW3020 diffractometer for the room-temperature individual XRD studies), TEM and SAED (FEI Titan), and ζ-potential and dynamic light scattering measurements (Malvern Zetasizer Nano). The second harmonic response was measured by probing the Hyper Rayleigh Scattering as previously reported^55^. Magnetic measurements were carried out with a custom built Vibrating Sample Magnetometer developed by the Magnetism and Spintronics group at TCD scanning from 1T to −1T as already described^59^.

### Colloidal suspension preparation

To measure the colloidal stability after annealing, the ζ-potential of BiFeO_3_ powder suspensions was analysed as a function of pH. The samples were prepared as recommended in the IUPAC report on the calculation of colloidal stability from electrophoretic mobility measurements: the ionic strength of the solutions was fixed at 10^−3^ M using NaCl. Five suspensions of BiFeO_3_ powders were prepared, setting the concentration of NaCl at 1 mM for the pH 7 solution, 0.99 mM for the pH 5 and 9 solutions and 0 mM for the pH 3 and pH 11 solutions. The pH was then adjusted to the target pH by adding drops of 0.1 M HCl or NaOH solution.

### Hyper Rayleigh Scattering measurements

Briefly, decanted suspensions of BiFeO_3_ were placed in the path of a vertically polarized laser of wavelength 1064 nm. A photomultiplier, set at 90° to the input, was used to detect the unpolarised scattered second harmonic light using a boxcar to gate at 532 nm.

Five solutions were prepared by dilution of the original concentration with 1 mM aqueous solution of NaOH and, for each of the five concentrations, the intensity was measured. The HRS intensity was plotted as a function of relative concentration and the nanoparticles’ effective hyperpolarisibility, <β_np_^2^>, was calculated from the linear portion of the curve from the eq. (1):

1 $${I}_{2\omega }=GN{T}_{np} < \,{\beta }_{np}^{2}\, > {I}_{\omega }^{2}$$

where G is an experimental constant and T_np_ is an internal field factor calculated from the solvent and nanocrystal refractive indices (here n_ω_ ∼2.76 and n_2ω_ ∼3.20 for BiFeO_3_)^7^.

The nanoparticle concentration N is estimated by preparing a larger volume sample in the same way as for HRS analysis, dispersing 10 mg of BiFeO_3_ in 1 L of 1 mM NaOH and decanting over three days. 950 mL of the supernatant was then evaporated in aliquots and weighed. Finally, the averaged SH coefficient <d> is calculated as from eq. (2), using V_np_ calculated from the DLS *Size by number* measurement as an estimate of the nanoparticle volume:

2 $$ < {\beta }_{np}\, > = < \,d\, > {V}_{np}$$

### Second Harmonic Microscopy Imaging

For optical nonlinear imaging Nikon A1R-MultiPhoton inverted microscope coupled with a Spectra-Physics Mai-Tai tunable oscillator (100 fs, 80 MHz, 700–1020 nm) was used. The spectral detection unit of the Nikon A1R system (32 channels, 400–650 nm detection range up to 32 channels, 400–650 nm detection range, three different gratings allowing 10, 6, or 2.5 nm resolution). In the plots in Fig. 5, we used the highest spectral resolution (2.5 nm) to fully resolve the second harmonic peak and 10 nm in the remaining spectral range.

## Electronic supplementary material

Supplementary Information
