# Author Correction: Preparation from a revisited wet chemical route of phase-pure, monocrystalline and SHG-efficient BiFeO_3_ nanoparticles for harmonic bio-imaging

**DOI:** 10.1038/s41598-019-43504-z

**Published:** 2019-10-30

**Authors:** Gareth Clarke, Andrii Rogov, Sarah McCarthy, Luigi Bonacina, Yurii Gun’ko, Christine Galez, Ronan Le Dantec, Yuri Volkov, Yannick Mugnier, Adriele Prina-Mello

**Affiliations:** 1 Department of Clinical Medicine, Trinity Translational Medicine Institute (TTMI), Trinity College Dublin, Dublin 8, Ireland; 2 CRANN Institute and AMBER centre, Trinity College Dublin, Dublin 2, Ireland; 3 Univ. Savoie Mont Blanc, SYMME, F-74000 Annecy, France; 4 GAP – Biophotonics, Université de Genève, 22 Chemin de Pinchat, CH-1211 Genève 4, Switzerland; 5 School of Chemistry, Trinity College Dublin, Dublin 2, Ireland; 6 Department of Histology, Cytology and Embryology, First Moscow State Sechenov Medical University, Moscow, Russian Federation

Correction to: *Scientific Reports* 10.1038/s41598-018-28557-w, published online 11 July 2018

The original version of this Article omitted an affiliation for Yuri Volkov. The correct affiliations for Yuri Volkov are listed below:

Department of Clinical Medicine, Trinity Translational Medicine Institute (TTMI), Trinity College Dublin, Dublin 8, Ireland

CRANN Institute and AMBER centre, Trinity College Dublin, Dublin 2, Ireland

Department of Histology, Cytology and Embryology, First Moscow State Sechenov Medical University, Moscow, Russian Federation

This has now been corrected in the HTML and PDF versions of this Article.

