# Supplementary material for: Preparation from a revisited wet chemical route of phase-pure, monocrystalline and SHG-efficient BiFeO3 nanoparticles for harmonic bio-imaging
Source: Sci Rep. 2018 Jul 11;8:10473. doi: 10.1038/s41598-018-28557-w (PMC6041297; doi:10.1038/s41598-018-28557-w)
Supplement: Supplementary file 1 — Supplementary Information [file 41598_2018_28557_MOESM1_ESM.pdf]

Supplementary Information

Preparation from a revisited wet chemical route of phase-pure, monocrystalline and SHG-efficient $BiFeO_3$ nanoparticles for harmonic bio-imaging

Gareth Clarke, Andrii Rogov, Sarah McCarthy, Luigi Bonacina, Yurii Gunko, Christine Galez, Ronan Le Dantec, Yuri Volkov, Yannick Mugnier and Adriele Prina-Mello

S1. Crystal structure of $BiFeO_3$

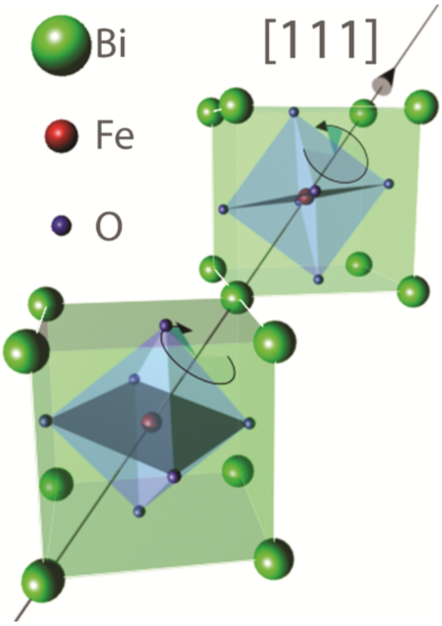

Figure S1 : Image of two $BiFeO_3$ primitive cells. $BiFeO_3$ is a rhombohedrally distorted perovskite in which oxygen octahedrons are rotated around the pseudocubic [111] axis as shown. 3D image created with Autodesk MAYA.

S2. Additional –OH groups increase crystallinity at lower temperatures

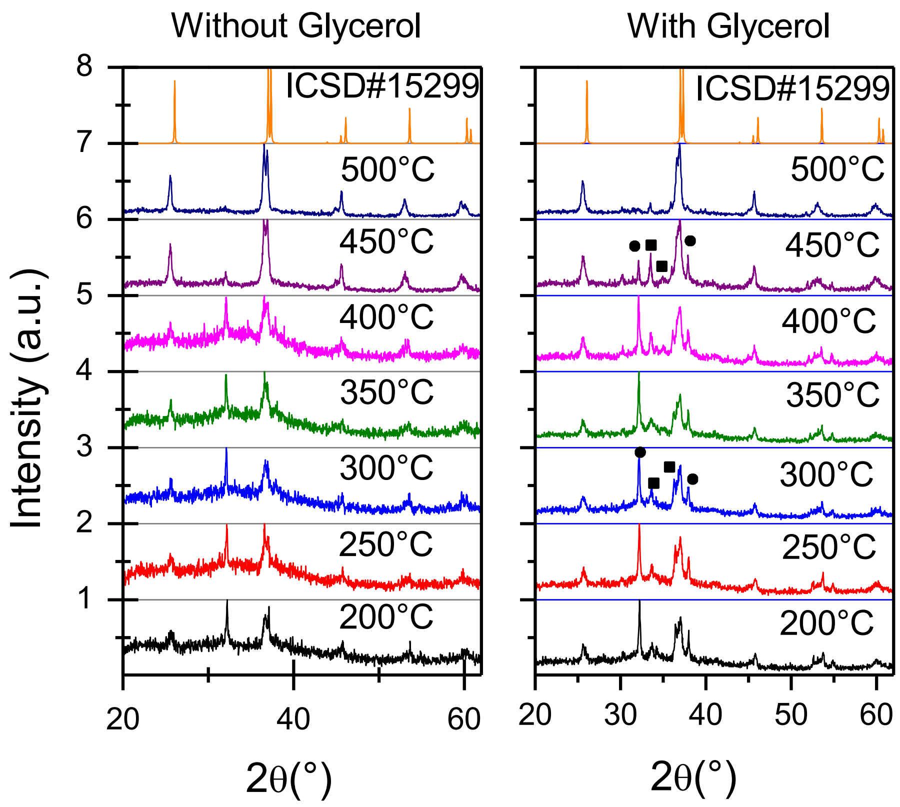

Figure S2: Temperature-resolved XRD patterns of powders prepared from mucic acid without (left) and with (right) glycerol. Left, although $BiFeO_3$ peaks are observed, there is a substantial amorphous background from 200 - 400°C. Right, from 200 - 400°C the background is less visible and the crystal peaks more prominent. At 300°C and 450°C, peaks denoted by ■ belong to the $Bi_{25}FeO_{39}$ phase whereas the extra peaks of varying intensity denoted by ● correspond very likely to an intermediary oxide phase that could not be determined.

S3. Improved crystallization enhances second harmonic properties

Size distribution by DLS

Sedimentation of residual aggregates was observed by time-resolved Dynamic Light Scattering (DLS), as well as by the change in the opaqueness of the sample over two days across the pH range investigated (the sample changed from yellow to transparent within the

pH range 3 - 9). Samples were therefore allowed to sediment for three days. At pH 11, the sample was still yellow after three days of sedimentation.
Dynamic Light Scattering measurements were carried out in aqueous solution at pH 11 using a Malvern Zetasizer Nano, before and after the sedimentation period as shown in Figure S3. These measurements were carried out to ensure that any aggregates could precipitate out of suspension, as the HRS intensity is a function of the crystallite size rather than of the aggregate size.

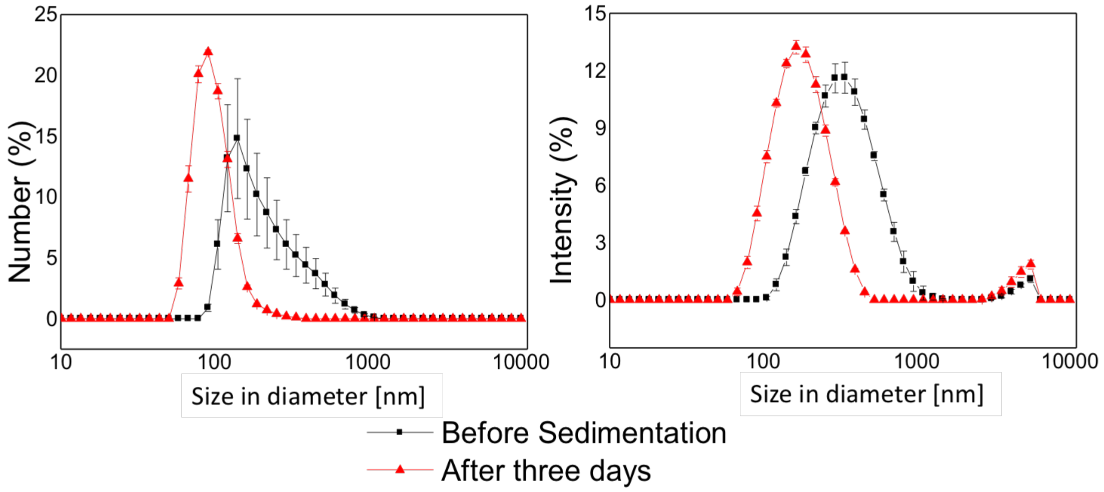

Sedimentation period = 3d		
Mean Size	Before	After
By Number	240nm	100nm
By Intensity	490nm	410nm
Z-average	320nm	180nm
PDI	0.23	0.26

Figure S3: DLS measurements of $BiFeO_3$ prepared via MA + NaCl, suspended in aqueous pH 11 before and after a sedimentation period of three days, showing the decrease in diameter (nm) by number (top left) and by intensity (top right), their mean sizes and the Z-average (table).

Colloidal stability of $BiFeO_3$ NPs in aqueous suspension

$BiFeO_3$ prepared via high concentrations of MA was not stable around pH 7; the absolute value of the ζ-potential was less than 30 mV, as can be seen in Figure S3.

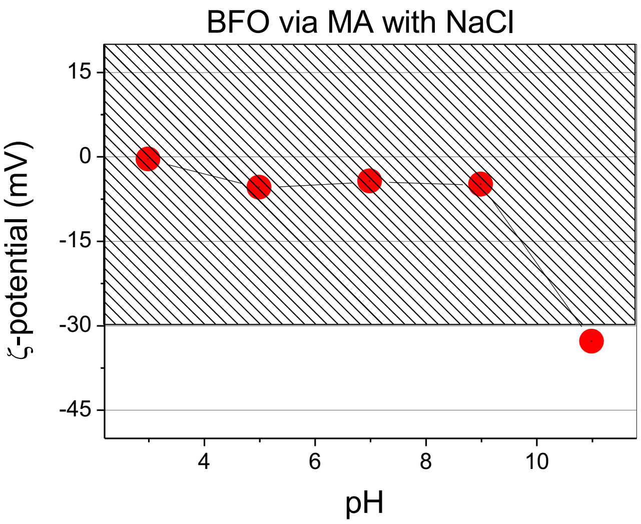

Figure S4: ζ-potential of $BiFeO_3$ NPs prepared with MA and NaCl as a function of pH. When the sample was prepared in the presence of salt the product was not stable at neutral pH as indicated with the dashed region

Hyper Rayleigh Scattering intensity *vs* relative concentration of $BiFeO_3$ in aqueous suspensions at pH 11

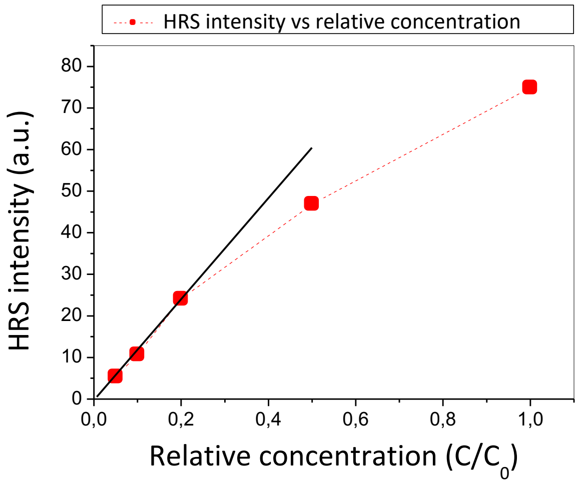

Figure S5: HRS intensity as a function of relative concentration. Only the lower, linear portion of the graph (black line) is used to calculate the effective hyperpolarisibility (and hence the second harmonic efficiency) because the deviation from linearity is considered to be due to absorption and multiple scattering in the sample.
